# Periglomerular afferent innervation of the mouse renal cortex

**DOI:** 10.3389/fnins.2023.974197

**Published:** 2023-01-26

**Authors:** Roman Tyshynsky, Sulagna Sensarma, Maureen Riedl, John Bukowy, Lawrence P. Schramm, Lucy Vulchanova, John W. Osborn

**Affiliations:** ^1^Graduate Program in Neuroscience, University of Minnesota, Minneapolis, MN, United States; ^2^Department of Surgery, University of Minnesota, Minneapolis, MN, United States; ^3^Department of Neuroscience, University of Minnesota, Minneapolis, MN, United States; ^4^Department of Electrical Engineering and Computer Science, Milwaukee School of Engineering, Milwaukee, WI, United States; ^5^Department of Biomedical Engineering, The Johns Hopkins University School of Medicine, Baltimore, MD, United States

**Keywords:** renal, afferents, glomeruli, juxtamedullary, renal cortex, interoception

## Abstract

Recent studies using a novel method for targeted ablation of afferent renal nerves have demonstrated their importance in the development and maintenance of some animal models of hypertension. However, relatively little is known about the anatomy of renal afferent nerves distal to the renal pelvis. Here, we investigated the anatomical relationship between renal glomeruli and afferent axons identified based on transient receptor potential vanilloid 1 channel (TRPV1) lineage or calcitonin gene related peptide (CGRP) immunolabeling. Analysis of over 6,000 (10,000 was accurate prior to the removal of the TH data during the review process) glomeruli from wildtype C57BL/6J mice and transgenic mice expressing tdTomato in TRPV1 lineage cells indicated that approximately half of all glomeruli sampled were closely apposed to tdTomato+ or CGRP+ afferent axons. Glomeruli were categorized as superficial, midcortical, or juxtamedullary based on their depth within the cortex. Juxtamedullary glomeruli were more likely to be closely apposed by afferent axon subtypes than more superficial glomeruli. High-resolution imaging of thick, cleared renal slices and subsequent distance transformations revealed that CGRP+ axons closely apposed to glomeruli were often found within 2 microns of nephrin+ labeling of glomerular podocytes. Furthermore, imaging of thick slices suggested that CGRP+ axon bundles can closely appose multiple glomeruli that share the same interlobular artery. Based on their expression of CGRP or tdTomato, prevalence near glomeruli, proximity to glomerular structures, and close apposition to multiple glomeruli within a module, we hypothesize that periglomerular afferent axons may function as mechanoreceptors monitoring glomerular pressure. These anatomical findings highlight the importance of further studies investigating the physiological role of periglomerular afferent axons in neural control of renal function in health and disease.

## 1. Introduction

Catheter-based renal nerve ablation (CBRNA) is an emerging therapy for treatment-resistant hypertension (Kiuchi et al., 2019). CBRNA indiscriminately destroys efferent (sympathetic) nerves that regulate renal vascular resistance, renin release and tubular sodium absorption, and afferent (sensory) nerves that modulate the activity of the sympathetic nervous system (Osborn and Foss, 2017). Although the anatomy and physiology of efferent renal nerves has been thoroughly studied and their theoretical role in the pathogenesis of diseases such as hypertension has been described for more than 75 years (Kottke et al., 1945), far less is known about the anatomy of afferent renal nerves and their roles in the neural control of the kidney or the modulation of the autonomic nervous system. For these reasons, the original rationale for CBRNA assumed that its effect was caused by ablation of efferent renal nerves. However, reports of improved glucose metabolism, decreased muscle sympathetic nerve activity, and fewer incidences of arrythmias following CBRNA led to the hypothesis that some of its beneficial effects were due to the ablation of renal afferent nerves that modulate efferent activity to several organs (Schlaich et al., 2009).

Our group has reported that afferent renal nerves play an instrumental role in the pathogenesis and maintenance of deoxycorticosterone acetate-salt (DOCA-salt) hypertension in rats. Employing a novel capsaicin-based method to selectively ablate renal afferent nerves expressing the transient receptor potential vanilloid 1 (TRPV1) channel (Foss et al., 2015; Banek et al., 2016), we reported that afferent renal denervation attenuated the development of hypertension in DOCA-salt rats by approximately 50%, an effect similar to that observed with total (efferent and afferent) denervation (Foss et al., 2015; Banek et al., 2016, 2018). These studies suggest that the diminished hypertension after total denervation in DOCA-salt rats is mediated by ablation of afferent, rather than efferent renal nerves. A recent study came to the same conclusion using the periaxonal method of afferent renal denervation in a mouse model of hypertension induced by unilateral renal artery stenosis (Ong et al., 2019).

Classically, renal afferent nerves have been identified using antibodies that target the afferent neurotransmitters calcitonin-gene-related-peptide (CGRP) and substance P (SP) (DiBona and Kopp, 1997). Those studies suggested that most afferent nerves reside in the wall of the renal pelvis rather than in the cortex or medulla. However, several reports exist of afferent axons in the renal cortex with brief descriptions of afferent axons closely apposed to all parts of the nephron, including the glomeruli (Barajas et al., 1984; Kurtz et al., 1988; Veelken et al., 2008; Ditting et al., 2009). Those studies reported SP + afferent axons accompanying the afferent arteriole and “entering [a] glomerulus” in rat (Ditting et al., 2009), the colocalization of CGRP and TRPV1 in the rat renal cortex (Ditting et al., 2009), and CGRP+ afferent axons “close to glomeruli” (Veelken et al., 2008). However, these descriptions of the anatomical relationship between glomeruli and afferent axons were not the primary focus of those studies, thus, a detailed quantitative analysis of the presence of afferent axons near glomeruli has not been reported. Although it is known that TRPV1 is a multimodal ion channel that responds to acidic stimulation, capsaicin, heat, hypertonicity, and mechanical stimulation (Caterina et al., 1997; Tominaga et al., 1998; Naeini et al., 2006; Ditting et al., 2009; Lin et al., 2015; Moore and Liedtke, 2017), its functional role in the kidney has not been fully elucidated.

Recent advances in tissue-clearing and imaging methods permit, for the first time, the rapid visualization of thick tissue sections (Matsumoto et al., 2019). We applied these methods to investigate the anatomical relationship between afferent axons and renal glomeruli in renal slices 150 μm or larger, thus allowing for the analysis of multiple glomeruli in the same image. Specifically, we combined the use of a transgenic mouse model, immunofluorescence, and advanced tissue clearing techniques to quantify the relationship between renal afferent nerves and glomeruli. To achieve this, we (1) developed a novel “glomerular scoring” method to visualize thousands of glomeruli in renal slices across the entire kidney and quantity the frequency of anatomical relationships between these glomeruli and axon subtypes, (2) determined the distance between afferent axons and glomeruli using high-resolution imaging, and (3) identified potential interglomerular interactions *via* common afferent axons using high volume imaging of cleared tissue. The results of this study reveal a previously undescribed anatomical relationship between cortical afferent renal nerves and glomeruli in the mouse kidney.

## 2. Materials and methods

### 2.1. Animals

All procedures were performed in accordance with the National Institutes of Health *Guide for the Care and Use of Laboratory Animals* and approved by the University of Minnesota Institutional Animal Care and Use Committee. The experiments were performed in 12–18-week-old male and female wildtype C57BL/6J mice and in transgenic mice expressing the fluorescent reporter protein tdTomato in a TRPV1-dependent manner. To generate these transgenic mice, homozygous mice expressing Cre under the control of the TRPV1 promoter (TRPV1-Cre mice; jax#017769) (Cavanaugh et al., 2011) were crossed with homozygous mice expressing a flox-stop-tdTomato reporter gene [Ai14 mice; jax#0079014 (Madisen et al., 2010)], directing the expression of tdTomato in TRPV1 + cells throughout development (TRPV1:Ai14). TRPV1 is expressed in primary afferent neurons more broadly during development and later downregulated (Cavanaugh et al., 2012). Thus, in our experiments, the expression of tdTomato was used as a proxy for afferent axon labeling, rather than as a reporter for the expression of TRPV1. This transgenic approach afforded greater sensitivity for visualization of afferent axons compared to traditional immunohistochemical techniques (Cavanaugh et al., 2011).

### 2.2. Immunohistochemistry for glomerular scoring analyses

Mice were deeply anesthetized using isoflurane and terminated *via* cardiac perfusion using ice cold calcium-free Tyrode’s solution (in mM: NaCl 116, KCl 5.4, MgCl_2_⋅6H_2_0 1.6, MgSO_4_⋅7H_2_O 0.4, NaH_2_PO_4_ 1.4, glucose 5.6, and NaHCO_3_ 26), followed by Lana’s fixative (4% paraformaldehyde and 0.2% picric acid in 0.1 M phosphate buffer pH 6.9). Kidneys were removed and stored in phosphate buffered saline (PBS). Immediately prior to sectioning, the kidneys were decapsulated and sectioned coronally on a Vibratome Series 1000 into 150 μm-thick sections, which were stored serially in PBS at 4°C until staining. For each animal, one section through the center of the kidney that included renal cortex, medulla, and pelvis was incubated in blocking buffer (PBS with 0.3% Triton-X100; 1% BSA, 1% normal donkey serum) at 4°C overnight and then incubated (2 days at 4°C) in primary antibodies diluted in blocking buffer (rabbit anti-DsRed, Takara Bio, San Jose, CA, USA, cat# 632496, 1:500; goat anti-nephrin, R&D Systems, Minneapolis, MN, USA, cat# AF3159, 1:500; sheep anti-TH, Millipore, Burlington, MA, USA, cat# AB1542, 1:500; rabbit anti-TH, Millipore, Burlington, MA, USA, cat# AB152, 1:500; rabbit anti-CGRP, Immunostar, Hudson, WI, USA, cat#24112, 1:500). Sections were washed 3 times for 30 min each in PBS at room temperature, followed by overnight incubation at room temperature in secondary antibodies (Cy3-conjugated donkey anti-rabbit, 1:300, cat# 711-165-152; Cy3-conjugated donkey anti-sheep, 1:300, cat# 713-165-147; Alexa 488-conjugated donkey anti-goat, 1:300, cat# 705-545-147; Cy5-conjugated donkey anti-rabbit, 1:300, cat# 711-175-152; Cy5-conjugated donkey anti-sheep, 1:300, cat# 713-175-147). The sections were then washed again, mounted on gelatin-coated slides, and allowed to adhere overnight at room temperature. Slide-mounted sections were dehydrated with increasing concentrations of ethanol (50, 75, 100, and 100% in diH_2_O, 30 min each), cleared in Xylenes (Macron Chemicals, cat#MAL8668) until the tissue appeared transparent, and finally coverslipped using DPX Mountant.

### 2.3. CUBIC clearing and immunohistochemistry protocol

Mice were deeply anesthetized using isoflurane and terminated *via* cardiac perfusion using ice cold calcium-free Tyrode’s solution, followed by 4% PFA (4% paraformaldehyde in phosphate buffered saline). The kidneys were post-fixed in 4% PFA for 24 h and then stored in PBS until decapsulation and sectioning. Sections (150 μm) were cleared using the CUBIC tissue clearing protocol as described by the manufacturer (Tissue-Clearing Reagent CUBIC-L (T3740) and CUBIC-R + (T3741), Tokyo Chemical Industry Co., Ltd., Portland, OR, USA). Briefly, the sections were washed in PBS 3 times for 2 h each at room temperature and incubated in 50% CUBIC-L in diH_2_O solution in a 37°C water bath for 24 h. The slices were then submerged in fresh 100% CUBIC-L solution and replaced in the hot water bath for 48 h, refreshing the solution after 24 h. Following washing in PBS 3 times for 2 h each, the slices proceeded through the staining protocol detailed below. Slices were incubated in diluent at room temperature for 24 h, followed by incubation in primary antibodies at room temperature for 48 h (goat anti-nephrin, R&D Systems, Minneapolis, MN, USA, cat# AF3159, 1:500; rabbit anti-CGRP, Immunostar, Hudson, WI, USA, cat#24112, 1:500; rabbit anti-TH, Millipore, Burlington, MA, USA, cat# AB152, 1:500). The samples were washed in PBS 3 times for 2 h each before incubation in secondary antibodies for 24 h at room temperature (1:300; Cy3-conjugated donkey anti-rabbit, 1:300, cat# 711-165-152; Alexa 488-conjugated donkey anti-goat, 1:300, cat# 705-545-147). Slices were then washed 3 times for 2 h each in PBS at room temperature, and then placed in a 50% CUBIC-R + in diH_2_O solution for 24 h at room temperature. Finally, slices were placed in 100% CUBIC-R + for at least 24 h, and stored at room temperature, protected from light until ready for imaging.

Just prior to imaging, the prepared slices were gently transferred to a 40 mm round #1.5 glass coverslip (Warner Instruments, Holliston, MA, USA, cat #64-1696) mounted in a circular chamber and submerged in CUBIC-R + medium. Two 0.1 mm thick, 40 mm round silicone gaskets (Bioptechs cat# 060319-2-0719) were inserted as spacers, and a second coverslip gently placed on top before the well was screwed shut. Care was taken to ensure no bubbles remained within the medium to prevent movement during long image collection periods.

### 2.4. Image collection

**FIGURE 1:**
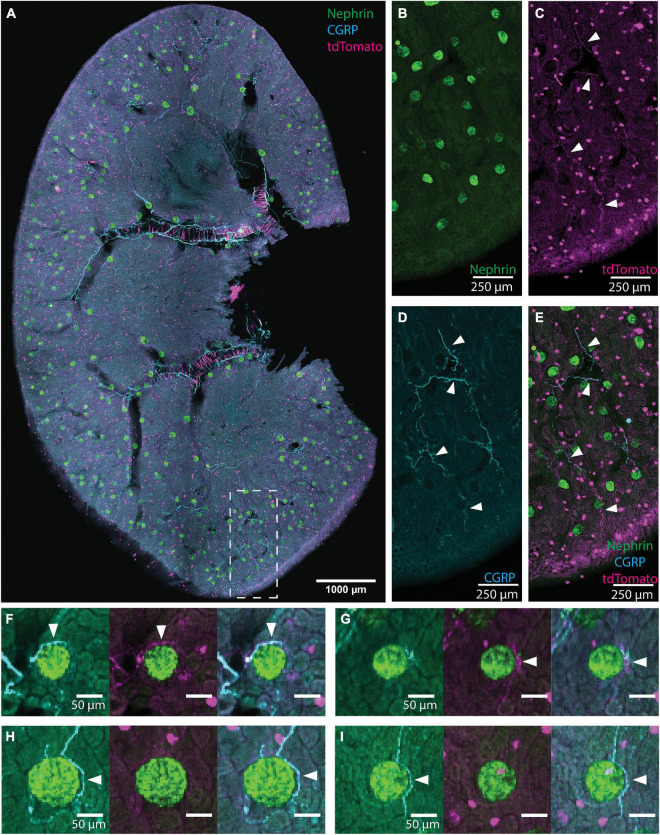
Illustration of glomerular scoring. **(A)** An image of native tdTomato fluorescence (magenta, tdTomato+ axons) and immunolabeling for nephrin (green, glomeruli) and calcitonin gene related peptide (CGRP) (cyan, peptidergic axons) in a renal slice from a TRPV1:Ai14 mouse. The image represents a maximum intensity projection of 6 optical sections collected 13.7 microns apart. While this image includes CGRP+ and tdTomato+ labeling for illustrative purposes, only images immunolabeled for one neuronal marker at a time were used during glomerular scoring. **(B–E)** A magnified inset from panel **(A)** illustrating the labeling of glomeruli and axons in the renal cortex (scale bars: 250 μm). **(B)** Nephrin+ labeling of glomerular podocytes. **(C)** Native tdTomato fluorescence. Examples of labeled axons are highlighted with arrowheads. The identity of globular cell-like profiles is unknown. They appeared to be associated with tubules and were not co-labeled with a pan-immune cell marker (anti-CD45, data not shown). They were clearly distinguished from axonal profiles and, therefore, did not affect the glomerular scoring. **(D)** CGRP+ labeling of afferent axons (some highlighted with arrowheads). **(E)** Composite image illustrating CGRP labeling and tdTomato fluorescence in afferent axons (some highlighted with arrowheads) in the renal cortex. **(F–I)** Examples of anatomical relationships between axons and glomeruli as observed while scoring individual glomeruli (scale bars: 50 μm). **(F)** Both CGRP+ and tdTomato+ axons (arrowheads) approach the glomerulus. **(G)** A tdTomato+ axon bundle (arrowhead) approaches the glomerulus, and although the cyan channel exhibits some labeling, its morphology is not consistent with the appearance of axons and is therefore not scored as an anatomical interaction between CGRP+ axons and the glomerulus. **(H)** A clear anatomical relationship between a CGRP+ axon bundle (arrowhead) and the glomerulus. Although there is faint labeling in the magenta channel, the signal to noise ratio is not high enough to count as a close apposition. **(I)** CGRP+ axon (arrowhead) surrounds the glomerulus extend beyond it. Scale bars: **(A)** 1000 μm, **(B–E)** 250 μm, **(F–I)** 50 μm.

Images of the sections prepared for glomerular scoring were taken with a Nikon A1R FLIM Confocal Microscope equipped with the A1R GaAsP Confocal system; objectives—Nikon Plan Apo 4×/0.2 NA (Figure 1); confocal aperture was set to software-determined auto settings. Sequential multi-fluorescent images were collected using 488 nm (Alexa488), 561 nm (Cy3), and 640 nm (cy5) laser excitation where appropriate, and collecting emission between 425 and 475 nm, 505–525 nm, and 550–650 nm, respectively. Images of the sections that underwent tissue clearing for high-resolution image collection were collected with a Nikon FN1 upright stand equipped with an A1R HP MP laser scanning head and a motorized Prior stage and piezo Z drive, controlled with NIS Elements 5.1 software; objectives—Nikon PlanApo LWD 25× water-immersion/NA 1.1 (Figures 3, 4), Nikon Fluor DIC M 40× water dipping/NA 0.8. The representative images presented in the figures were adjusted for brightness, contrast, and color using Fiji software.

### 2.5. Analysis of whole-slice, low-resolution images

Images of renal slices were used to determine the prevalence of periglomerular axons in a process referred to here as “glomerular scoring.” Although parameters were adjusted individually for each renal slice depending largely on whether the slice laid flat on the slide, most slices were imaged as 5–15 optical sections with a z-step between 5 and 15 μm. These images were processed into multiple maximum intensity projections of 2–6 optical sections. Care was taken to ensure that one individual glomerulus was not scored multiple times between separate projections. The projections were then processed using a MATLAB script that localized immunofluorescently labeled glomeruli and stores their locations in a cell array (Bukowy et al., 2018). Filtering parameters were adjusted on an image-by-image basis to maximize the number of glomeruli detected in each stack while minimizing false positives. One by one, images of individual glomeruli were then displayed at 2.5× zoom and split into individual channels and a composite image. The user was prompted about the presence of an axon closely apposed to the nephrin+ labeling. A glomerulus was scored as having an axon “closely apposed” if an axon appeared within four pixels of the nephrin+ labeling (images were collected at 2.34 microns per pixel resolution). Only fine fiber-like structures were identified as axons and considered for scoring. The result of this analysis was a matrix indicating whether a glomerulus was present in the sampled image, whether it was closely apposed by an axon, its depth within the cortex, and its x and y coordinates. For each slice, the resulting matrices from each z-stack were concatenated to result in a single matrix representing all glomeruli sampled from a single renal slice.

Based on previously published methods (Bukowy et al., 2018), glomeruli were automatically categorized as juxtamedullary, midcortical, or superficial by the MATLAB script. As glomeruli were identified, their depths within the cortex were recorded. The standard deviation of the depths of glomeruli present within a single renal slice was multiplied by three to estimate the corticomedullary border of the slice. This approximation of the depth of the renal cortex was visually confirmed for each slice. The depth of the cortex was then binned into three categories: the deepest 1/4 being “juxtamedullary,” the most superficial 1/4 being “superficial,” and the middle 1/2 being “midcortical,” based on classical glomerular anatomical characterizations using depth (Kriz et al., 1988). These categories were then assigned to each glomerulus based on its depth within the cortex and used for parcelation of overall results based on glomerular category.

For all glomerular scoring experiments, scoring was performed by two independent researchers. The percent of glomeruli scored as having a closely apposed axon was determined from the concatenated matrix representing each slice, the result of which was then averaged across observers to result in the reported overall scores.

### 2.6. Analysis of high-resolution images of cleared tissue

The distances between CGRP+ labeling and nephrin+ labeling in CUBIC-processed renal slices from 7 animals (4F, 3M) were estimated using both 3-D and 2-D analysis methods. For each animal, 2 glomeruli were sampled from each of the superficial, midcortical, and juxtamedullary categories to a total of 6 samples per animal, and a total of 42 glomeruli. Images of whole glomerular volumes at 0.16 μm/px resolution were captured as described above. 3-D models of the nephrin+ labeling and CGRP+ axon bundles were created using the Surfaces Wizard function within Imaris 9.8. A distance transformation was then computed from each glomerulus to determine the shortest distance between each CGRP+ axon and the glomerulus. Images whose 3-dimensional view did not lend itself to accurate modeling were instead analyzed in 2-dimensions using Fiji software. Here, images were loaded as a composite of the nephrin and CGRP labeling and their contrast increased to better visualize CGRP+ and nephrin+ labeling. Advancing through the entire volume of the image, two adjacent optical sections at a time were stacked into a maximum intensity projection, and the closest distance between CGRP+ and nephrin+ labeling for each projection was recorded using the line tool. For either analysis technique, care was taken to ensure that only nephrin+ or CGRP+ labeling of high intensity were sampled for distance measurements. Thus, the halo-like low-intensity fluorescence surrounding nephrin+ labeling in some images was not considered when measuring distances between CGRP+ and nephrin+ labeling. The minimum distance between CGRP+ and nephrin+ labeling is reported for each glomerulus. The dataset shown in Figure 3 includes both 3-D and 2-D measurements.

In addition to the minimum distance measurements between CGRP+ and nephrin+ labeling described above, estimations of the distance between CGRP+ axons and Bowman’s space were made from high-resolution images of cleared tissue. To do so, the FIJI line tool was used to measure the minimum distance between an axon and the halo-like fluorescence surrounding nephrin+ labeling for the observed closest axon in every other optical section that contained the axon labeling. An estimate of the length of the axon that traveled along Bowman’s space was then also made using the FIJI line tool.

### 2.7. Statistical analysis

One-way ANOVAs with multiple comparisons were performed to investigate the differences between the presence of different molecular markers near glomeruli, and repeated measures (RM) one-way ANOVAs with multiple comparisons were employed to investigate the difference between axon relationships with glomeruli of different categories by depth within the same animal.

## 3. Results

### 3.1. Distribution of CGRP+ and tdTomato+ afferent axons near glomeruli

**FIGURE 2:**
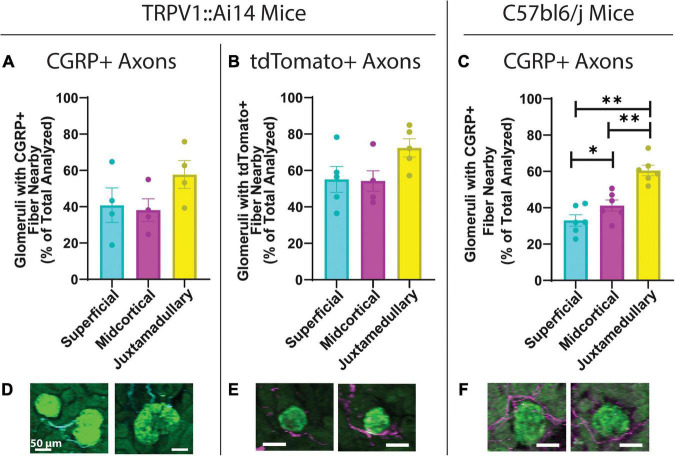
Quantitative analysis of the anatomical relationship between afferent axons and glomeruli in TRPV1:Ai14 and wildtype C57BL/6J mice. **(A)** Prevalence of calcitonin gene related peptide (CGRP)+ afferent axons near glomeruli in TRPV1:Ai14 mice (*n* = 4), presented by glomerular category based on depth within the cortex. Glomeruli at all depths were found to have similar frequencies of anatomical interactions with CGRP+ axons in TRPV1:Ai14 mice (Superficial: 40.78 ± 9.50%, Midcortical: 38.09 ± 6.29%, Juxtamedullary: 57.74 ± 7.70%; total number of evaluated glomeruli: 1346, 1 slice per animal). **(B)** Prevalence of tdTomato+ afferent axons near glomeruli in TRPV1:Ai14 mice (*n* = 5), presented by glomerular category based on depth within the cortex. Glomeruli at all depth categories were found to have similar frequencies of anatomical interactions with tdTomato+ axons in TRPV1:Ai14 mice (Superficial: 55.14 ± 7.06%, Midcortical: 54.22 ± 5.59%, Juxtamedullary: 72.40 ± 4.98%; total number of evaluated glomeruli: 1476, 1 slice per animal). **(C)** Prevalence of CGRP+ afferent axons near glomeruli in wildtype C57bl6/j mice (*n* = 6), presented by glomerular category based on depth within the cortex. Deeper glomeruli had closely apposed CGRP+ axon bundles more frequently than more superficial glomeruli (Superficial: 33.00 ± 3.11%, Midcortical: 41.22 ± 3.05%, Juxtamedullary: 60.57 ± 2.90%; total number of evaluated glomeruli: 3532, 1 slice per animal). **(D–F)** Examples of anatomical relationships between CGRP+ axons and glomeruli in TRPV1:Ai14 mice **(D)**, tdTomato+ axons and glomeruli in TRPV1:Ai14 mice **(E)**, and CGRP+ axons and glomeruli in C57bl6/j wildtype mice **(F)** as presented while scoring individual glomeruli (scale bars: 50 μm). (Mean ± SEM, *p < 0.05, ^**^p < 0.01 by Repeated Measures One-Way ANOVA with multiple comparisons).

To determine the frequency by which neuronal axons or axon bundles were found near renal glomeruli, a custom-designed MATLAB script was created to semi-automatically localize glomeruli labeled with a nephrin antibody and display them individually for assessment of the presence of closely apposed axon bundles. Figure 1A shows an example of a tiled image of an immunolabeled TRPV1:Ai14 mouse renal slice identifying nephrin (glomeruli, green), CGRP+ axons (cyan), and the endogenous fluorescence of tdTomato+ axons (magenta). Panels 1B–1E present a portion of renal cortex with images of individual labeling and a composite image. Examples of individual glomeruli presented during the scoring process are shown in Figures 1F–I. It is important to note that although Figure 1 depicts an image of a renal slice showing three channels, analyzed images were only labeled for nephrin and a single neuronal marker. This ensured that analyses of tdTomato expression were done with antibody-amplified labeling of tdTomato, avoiding underrepresentation of tdTomato due to weak endogenous fluorescence. During the scoring process, the scorer was presented with the image of nephrin labeling, as well as a composite images of the nephrin labeling with the axon labeling. Examples of composite images presented to the scorer are shown in Figures 2D–F. These experiments were performed in both transgenic TRPV1:Ai14 mice and wildtype C57BL/6J mice.

**FIGURE 3:**
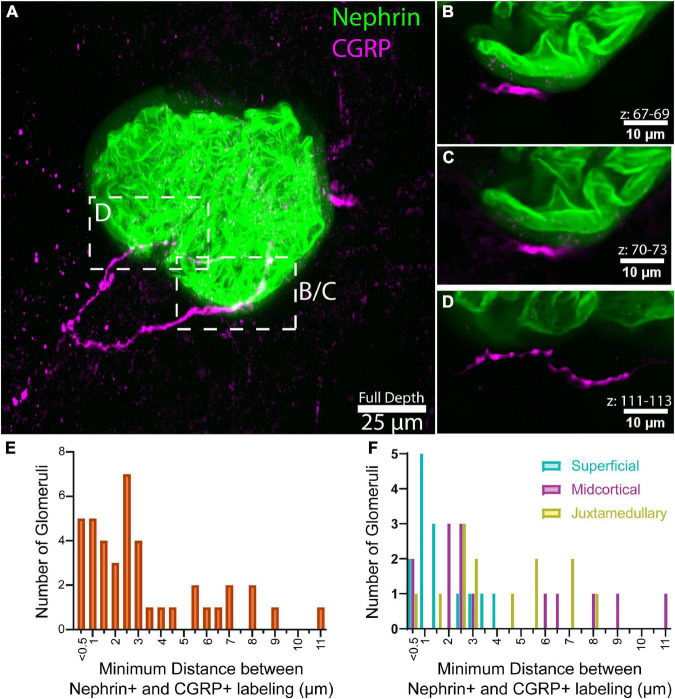
Analysis of close appositions between glomeruli and afferent axons. **(A)** Maximum intensity projection of 136 optical sections, collected 0.65 μm apart, total volume 88.4 μm. **(B–D)** Maximum intensity projections of subvolumes of A depict close appositions between calcitonin gene related peptide (CGRP)+ axons and nephrin labeling. Optical section numbers included in each projection are indicated above the scale bars. CGRP labeling does not overlap with the halo-like fluorescence surrounding nephrin labeling (faint green), suggesting that the axons do not penetrate below Bowman’s capsule, but are immediately apposed to it. Panels **(B,C)** illustrate an approximately 11.2 μm-long segment of CGRP labeling located 0.4–1.3 μm from the halo-like fluorescence surrounding nephrin labeling. **(E)** Distribution of the minimum distance between nephrin and CGRP labeling in high-resolution images of 42 glomeruli (*n* = 7 animals, 6 glomeruli per animal). **(F)** Minimum distance between nephrin and CGRP labeling by glomerular category, displayed as overlay [*n* = 7 animals, 2 glomeruli per category each (42 total)]. Scale bars: **(A)** 25 μm, **(B–D)** 10 μm.

**FIGURE 4:**
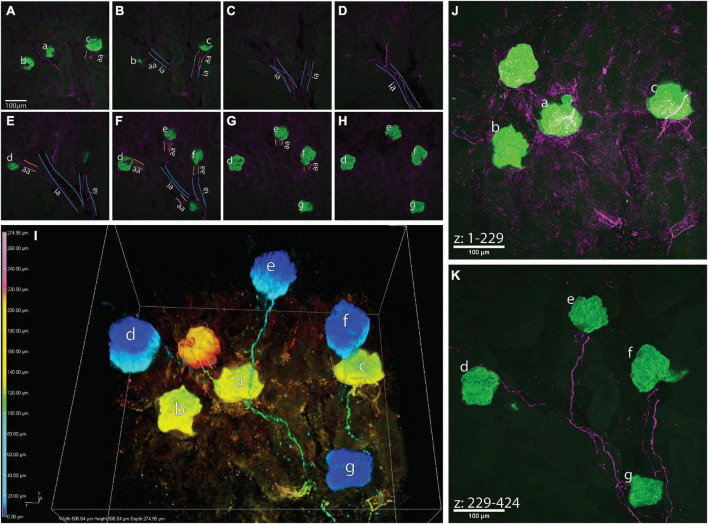
A high-resolution image of calcitonin gene related peptide (CGRP)+ bundles approaching multiple glomeruli. **(A–H)** Maximum z-projections of 25 optical sections, collected 0.65 μm apart, total subvolumes of 16.25 μm each illustrating common interlobular arteries (ia, outlined in blue) branching into subsequent afferent arterioles (aa, outlined in yellow) before supplying glomeruli (a–g, green, nephrin+). CGRP+ bundles (purple) follow the branching of the common interlobular arteries and extend to glomeruli a–g as a module. It appears that glomerulus f originates from another interlobular artery, which may share a common origin with the interlobular artery giving rise to glomeruli a–e and g. **(I)** 3-Dimensional representation of connectivity of glomeruli by CGRP+ bundles. Color-coded by depth within the image (warm tones: deep in image, cool tones: shallow in image), nephrin-labeled glomeruli (globular structures) appear “connected” *via* CGRP-labeled axons, which largely travel along shared interlobular arteries en route to glomeruli. **(J,K)** Maximum intensity projections of z-stacks splitting the depth of the image into two sub-volumes [**(J)** 148 μm, **(K)** 127 μm]. Glomeruli represented as warm tones in panel **(I)** (a–c) are captured within the first 229 optical sections of the image, and glomeruli represented as cool tones in panel **(I)** (d–g) are present in optical sections numbering 229–424. Scale bars: (**A–H,J,K**), 100 μm.

The distribution of peptidergic afferent axons near glomeruli was determined by immunolabeling both wildtype and TRPV1:Ai14 mouse kidneys for calcitonin gene-related peptide (CGRP). On average, in TRPV1:Ai14 animals, 43.34 ± 6.82% of the 1346 glomeruli inspected were accompanied by a closely apposed CGRP+ axon or axon bundle across 4 animals. In 6 wildtype mice, 48.3 ± 1.4% of the 3532 glomeruli analyzed were closely apposed by a CGRP+ axon or bundle. Analysis of immunofluorescently amplified tdTomato+ axons in TRPV1:Ai14 animals (1476 glomeruli analyzed across 5 mice) indicated that about half of the glomeruli had a tdTomato+ axon in close proximity (58.49 ± 5.3%).

To determine whether the frequency of close apposition of glomeruli and afferent axons varied across the depth of the renal cortex, the depths of glomeruli analyzed in the high-throughput glomerular scoring were collected for further analysis. Based on previously described mathematical approximations of cortical depths (Kriz et al., 1988; Bukowy et al., 2018), glomeruli were categorized as superficial (SF), midcortical (MC), or juxtamedullary (JM). Figures 2A, B depicts that in TRPV1:Ai14 mice, for both CGRP+ and tdTomato+ axons, there was a trend toward more frequent apposition to juxtamedullary glomeruli (CGRP+, Figure 2A: 40.78 ± 9.5% for SF, 39.09 ± 6.29% for MC, 57.74 ± 7.69% for JM, RM one-way ANOVA, *p* = 0.0581; tdTomato+, Figure 2B: 55.14 ± 7.06% for SF, 54.22 ± 5.59% for MC, 72.40 ± 4.98% for JM, RM one-way ANOVA, *p* = 0.0670). Figure 2C illustrates that in wildtype C57BL/6J mice, juxtamedullary glomeruli were the most likely to have a closely apposed CGRP+ axon or bundle, followed by midcortical glomeruli and finally superficial glomeruli (SF: 33.00 ± 3.11%; MC: 41.22 ± 3.05%; JM: 60.57 ± 2.90%, RM one-way ANOVA with multiple comparisons, *p* = 0.0006; SF:MC *p* = 0.0143; SF:JM *p* = 0.0027; MC:JM *p* = 0.0098). Figures 2D–F depicts examples of the composite images presented during the scoring process.

### 3.2. High-resolution evaluation of the apposition of afferent axon bundles to glomerular nephrin+ labeling

Although the previous experiments investigated the relationship between renal glomeruli and axon subtypes by sampling thousands of glomeruli from images of whole renal slices, those low-resolution images did not allow more in-depth queries into these relationships. Thus, we utilized the CUBIC passive tissue clearing method to capture higher resolution images (0.16 μm/px) of entire glomerular volumes for a more accurate determination of distances between glomeruli and axon subtypes (Figures 3A–D). Imaris and FIJI software were used to determine the minimum distance between each CGRP+ axon bundle and nephrin+ labeling for each glomerulus (*n* = 42 glomeruli from 7 mice–2 juxtamedullary, 2 midcortical, and 2 superficial from each animal).

Figure 3E shows that for 28 (66%) of the sampled glomeruli, the minimum distance between CGRP+ axons and nephrin+ labeling was less than 3 μm (median: 2.55 μm). Furthermore, Figure 3F illustrates that the minimum distance appeared to be shorter for superficial glomeruli compared to midcortical and juxtamedullary glomeruli (SF: 1.722 ± 0.289 μm; MC: 4.179 ± 0.911 μm; JM: 3.7658 ± 0.653 μm).

Assuming that the halo-like fluorescence surrounding the nephrin+ labeling delineates Bowman’s space, we did not observe concrete evidence that the CGRP+ axon bundles penetrated Bowman’s capsule. However, as shown in Figures 3B, C, CGRP+ axons were seen to follow within approximately 1 μm of the nephrin halo for approximately 11.2 μm of axon length. This 1 μm distance probably represented the location of the parietal epithelial cells of Bowman’s capsule. Such close apposition of CGRP+ axons and Bowman’s space was observed in 23 of the glomeruli in our sample. In most instances, the axons traveled within <2 um of the nephrin+ halo for more than 10 μm of axon length, with some axons remaining near Bowman’s space for up to 70 μm of their length. Instances were also observed in which there was no measurable distance between the CGRP+ labeling and the presumed Bowman’s space, indicating that the axon may have been adjacent to the surface of Bowman’s capsule, or potentially inside the surface of the capsule. However, higher resolution imaging techniques would be required to fully elucidate this relationship. No observable differences in CGRP+ axon distance from Bowman’s space were noted between juxtamedullary, midcortical, and superficial glomeruli.

### 3.3. CGRP+ bundles approach multiple glomeruli that share the same interlobular artery

Figure 4 depicts an example of a large-volume image of cleared a kidney slice that captured glomeruli sharing vascular input from a common interlobular artery. Although the vasculature was not labeled in these experiments, the autofluorescent background labeling within the samples depicted in Figures 4A–H (and lack thereof, indicating lumen) allowed for the approximate visualization of vascular structures. CGRP+ bundles (magenta) traveled along the vasculature, following the branching of an interlobular artery (ia) into subsequent afferent arterioles (aa), and eventually approaching multiple glomeruli (green, nephrin) (a-g). It remains to be determined whether this anatomical relationship between afferent axons and modules of glomeruli that share a common interlobular artery reflects shared sensory innervation of the glomeruli.

## 4. Discussion

To our knowledge, this is the first study to employ advanced anatomical methods to characterize the afferent innervation of the cortex of the mouse kidney. The major finding is the presence of afferent axons identified based on TRPV1 lineage or CGRP immunolabeling in close apposition to glomeruli. The significance of this finding is discussed below in terms of (1) novel anatomical findings, (2) the hypothetical modality and function of periglomerular afferent axons, and (3) the potential role of these renal afferent nerves in physiology and pathophysiology.

Several caveats regarding our visualization of cortical afferent axons should be noted. First, although we report a trend toward a higher presence of tdTomato+ compared with CGRP+ afferent axons near glomeruli, this trend is likely due to the transgenic expression of tdTomato in afferent neurons that expressed TRPV1 during development but not in maturity. While during development TRPV1 is expressed in both peptidergic and non-peptidergic neurons (Guo et al., 2001; Cavanaugh et al., 2012), in adult mice, unlike rats, TRPV1 is almost exclusively localized to peptidergic afferent neurons, with very little expression in IB_4_-binding non-peptidergic neurons (Zwick et al., 2002; Price and Flores, 2007). This developmental regulation of TRPV1 expression is not captured in the TRPV1:Ai14 mouse line used here, and the tdTomato+ renal afferent axons likely belong to both peptidergic and non-peptidergic primary afferent neurons. Second, although both anatomical and physiological studies show that CGRP+ or TRPV1 + axons represent common renal afferent subtypes, we cannot rule out the existence of renal cortical afferent axons that were not visualized in our study [for example, myelinated axons (Simon and Schramm, 1984; Knuepfer and Schramm, 1987)]. Despite these potential limitations, we report several novel findings.

### 4.1. Periglomerular afferent axons likely contact Bowman’s capsule

To the best of our knowledge, this study represents the first attempt to quantify the anatomical relationship between glomeruli and CGRP+ axons or afferent axons identified using a transgenic approach. In both TRPV1:Ai14 and wildtype mice, approximately 40% of the analyzed glomeruli were closely apposed by CGRP+ axons. Additionally, in TRPV1:Ai14 mice, approximately half of the glomeruli analyzed were closely apposed by tdTomato+ afferent axons. Additional experimental approaches would be necessary to definitively determine whether these close anatomical relationships constitute innervation of glomerular structures.

Our high-resolution imaging and analyses of the minimum distances between CGRP+ axon bundles and the nephrin+ labeling of glomerular podocytes indicate that afferent axons often travel within a few microns of nephrin+ labeling. At present, however, our data do not suggest that periglomerular afferent axons enter Bowman’s capsule. During analysis, special attention was given to ensure that the halo-like labeling surrounding nephrin+ labeling in some samples was not included in distance measurements between glomeruli and CGRP+ afferent axons. This halo-like labeling provides an estimation of Bowman’s space in the absence of direct labeling of the parietal epithelial cells of Bowman’s capsule. Based on a lack of observed overlap between CGRP+ labeling and this halo-like labeling in individual optical sections, we conclude that periglomerular afferent axons probably do not penetrate Bowman’s capsule, but rather travel along its surface. For example, Figures 3B, C illustrate an approximately 11 μm length of a CGRP+ axon that remains within 0.4–1.3 μm from the halo-like fluorescence surrounding the nephrin+ labeling that we assume represents Bowman’s space. Measurements of this relationship between CGRP+ axons and Bowman’s space in additional high-resolution images of glomeruli indicate that close proximity between the structures is common, often for greater than 10 μm of axon length and, in some cases, for up to 70 μm. Additional experiments in which the parietal epithelial cells constituting Bowman’s capsule are labeled and which employ even higher resolution imaging will be necessary to investigate these close anatomical relationships more fully.

### 4.2. TRPV1 and CGRP expression in periglomerular afferent axons suggest potential modalities

TRPV1 + and/or CGRP+ expressing axons subserve a variety of modalities that are specific to most organs and tissues. Known stimuli include (but are not limited to) mechanical force, capsaicin, heat, hypertonicity, cytokines, pH, anandamide, lipoxygenase products of arachidonic acid, and N-arachidonoyl dopamine (Van Der Stelt and Di Marzo, 2004; Sharif-Naeini et al., 2008; Dhaka et al., 2009; Lin et al., 2015; Moore and Liedtke, 2017). The present experiments did not address the potential modalities subserved by TRPV1 + and CGRP+ renal afferents, nor did they aim to determine the co-expression of tdTomato and CGRP within a single fiber. Nevertheless, their close proximity to glomeruli suggests that they may be mechanoreceptors, a modality that has been well documented in renal nerves (Feng et al., 2008; Li and Wang, 2008; Lin et al., 2015).

### 4.3. Potential functional significance of mechanosensitive periglomerular afferent axons near individual glomeruli

Glomerular capillary pressure, which determines blood flow into and out of the glomerulus, is a function of the vascular resistance (i.e., diameter) of the afferent and efferent arterioles, respectively. Glomerular diameter oscillates with beat-to-beat changes in renal arterial pressure as observed by *in vivo* imaging in the rat (Peti-Peterdi et al., 2015). As such, mechanosensitive axons surrounding Bowman’s capsule may be responsive to glomerular pressure in a manner similar to the pulse synchronous activity of arterial baroreceptors, which also express TRPV1 channels. Since afferent axons were found within 3 μm of the nephrin+ labeling of podocytes and less than 1 μm from Bowman’s space, these afferents are well located to sense changes in glomerular diameter. Although our studies did not directly label Bowman’s capsule, we found no evidence of afferent axons penetrating Bowman’s capsule, suggesting that the function of periglomerular afferent axons is probably not related to a chemosensory role in sensing filtrate composition directly within the corpuscle.

### 4.4. Hypothetical significance of mechanoreception of “Modules” of glomeruli

The 3-dimensional image in Figure 4 shows multiple glomeruli whose afferent arterioles were traced to a common interlobular artery. This interconnected “module” of glomeruli is in close apposition to one to two bundles of CGRP+ axons and their branches. Importantly, one CGRP+ bundle visualized in these experiments may represent an individual neuron’s axon that sends projecting branches to downstream structures, or a bundle of individual primary afferent axons that independently branch off the bundle to their targets. If glomeruli within a module are apposed by branches of a single axon, this anatomical relationship may underlie integration of sensory signals from individual branches in a common afferent axon. For example, mechanotransduction at an individual glomerulus may not be sufficient to generate depolarizations that exceed a primary afferent neuron’s threshold to generate an orthodromic action potential. However, a module of glomeruli with similar pressures (Holstein-Rathlou, 1987) would depolarize several branches of a single afferent axon that then summate to exceed the threshold for firing an action potential.

What is the physiological response to activation of periglomerular renal afferent nerves? Further studies are needed to address this question, but the responses to non-specific afferent renal nerve stimulation have been investigated. Increased afferent renal nerve activity has been shown to have sympathoexcitatory effects in both pathological and healthy conditions (Faber and Brody, 1985; Stella et al., 1987). Thus, mechanosensation of glomerular pressure changes, including changes to modules of glomeruli, could initiate a reflex arc to regulate glomerular filtration. For example, increased glomerular pressure would stimulate sympathoexcitatory afferent renal nerves, resulting in increased renal efferent nerve activity, ultimately resulting in afferent arteriolar vasoconstriction *via* the release of norepinephrine and a normalization of glomerular pressure.

### 4.5. Possible pathological relevance of periglomerular afferent axons

In addition to the orthodromic activation of central neural pathways and reflex arcs, the antidromic propagation of depolarizations within branches of primary afferents and local release of signaling mediators could also have effects on renal homeostasis. Of particular interest is the expression of CGRP by periglomerular afferent axons. The release of neuropeptides such as CGRP from dense core vesicles typically requires higher calcium influx into the terminal than the release of neurotransmitters from small synaptic vesicles. It is possible that renal inflammation, seen in some models of hypertension and other renal diseases, may lead to the sensitization of periglomerular afferent axons, causing previously insufficient signals to result in the antidromic release of CGRP.

The antidromic release of CGRP in the renal cortex may lead to different effects on renal hemodynamics. As a potent vasodilator, CGRP causes a dose-dependent alleviation of vasoconstriction of afferent arterioles induced by both pressure and angiotensin II, but it does not affect efferent arteriole diameter (Edwards and Trizna, 1990; Reslerova and Loutzenhiser, 1998). *Via* this mechanism, the antidromic release of CGRP by periglomerular afferent axons may, therefore, function in part to vasodilate the afferent arteriole and, consequently, increase glomerular pressure. In contrast, CGRP in the renal cortex can act as a stimulator of renin secretion by the juxtaglomerular apparatus (Kurtz et al., 1988). *Via* the renin-angiotensin system, this CGRP-mediated stimulation of renin release can have a multitude of downstream effects, including vasoconstriction and increased tubular reabsorption (Castrop et al., 2010). Thus, although the effects of CGRP release by periglomerular afferent axons are difficult to predict, it may nonetheless serve an important role in maintaining renal homeostasis *via* a neural mechanism, even in pathological states such as renal inflammation.

## 5. Conclusion

These studies highlight the prevalence of afferent axons near glomeruli in the mouse renal cortex. Moreover, CGRP+ afferent axons often travel within 3 μm of the nephrin+ labeling of podocytes and within 1 μm of the halo-like fluorescence surrounding nephrin+ labeling, which is presumed to represent Bowman’s space, and likely comes in direct contact with Bowman’s capsule. Finally, afferent axon bundles are shown to branch to travel in close proximity to multiple glomeruli within a module, suggesting a common source of neurovascular control for glomeruli that share the same interlobular artery. Taken together, these data suggest that afferent axons play important roles by monitoring glomerular pressures through a mechanosensitive mechanism and are likely influential in the regulation of glomerular pressures and filtration rate in both disease and healthy states. Furthermore, these proposed mechanisms would not require the presence of mechanosensitive axons near every glomerulus–sampling from a subset of glomeruli would be sufficient to elicit effects on a wider population of glomeruli either *via* a more global sympathoexcitatory response or *via* the release of vasoactive mediators. Further investigations of these periglomerular afferent axons are needed to support or refine this hypothesis and discern their precise physiological function to advance the field’s understanding of the role of renal nerves in normal and pathological states.

## Data availability statement

The raw data supporting the conclusions of this article will be made available by the authors, without undue reservation.

## Ethics statement

The animal study was reviewed and approved by University of Minnesota Institutional Animal Care and Use Committee.

## Author contributions

RT, LV, and JO conceptualized the project. RT and SS were responsible for data collection and analysis. MR was involved in tissue collection and immunohistochemical labeling. JB provided foundational MATLAB scripts upon which final analyses were built. RT, LS, LV, and JO participated in the preparation of the manuscript. All authors contributed to the article and approved the submitted version.
